# Metabolic Response to Heat Stress in Late-Pregnant and Early Lactation Dairy Cows: Implications to Liver-Muscle Crosstalk

**DOI:** 10.1371/journal.pone.0160912

**Published:** 2016-08-11

**Authors:** Franziska Koch, Ole Lamp, Mehdi Eslamizad, Joachim Weitzel, Björn Kuhla

**Affiliations:** 1 Institute of Nutritional Physiology “Oskar Kellner”, Leibnitz Institute for Farm Animal Biology (FBN), Dummerstorf, Germany; 2 Schleswig Holstein Chamber of Agriculture, Department of Animal production, Futterkamp, Blekendorf, Germany; 3 Department of Animal Science, Campus of Agriculture and Natural Resources, University of Tehran, Tehran, Iran; 4 Institute of Reproductive Biology, Leibnitz Institute for Farm Animal Biology (FBN), Dummerstorf, Germany; University of Edinburgh, UNITED KINGDOM

## Abstract

Climate changes lead to rising temperatures during summer periods and dramatic economic losses in dairy production. Modern high-yielding dairy cows experience severe metabolic stress during the transition period between late gestation and early lactation to meet the high energy and nutrient requirements of the fetus or the mammary gland, and additional thermal stress during this time has adverse implications on metabolism and welfare. The mechanisms enabling metabolic adaptation to heat apart from the decline in feed intake and milk yield are not fully elucidated yet. To distinguish between feed intake and heat stress related effects, German Holstein dairy cows were first kept at thermoneutral conditions at 15°C followed by exposure to heat-stressed (HS) at 28°C or pair-feeding (PF) at 15°C for 6 days; in late-pregnancy and again in early lactation. Liver and muscle biopsies and plasma samples were taken to assess major metabolic pathway regulation using real-time PCR and Western Blot. The results indicate that during heat stress, late pregnant cows activate Cahill but reduce Cori cycling, prevent increase in skeletal muscle fatty acid oxidation, and utilize increased amounts of pyruvate for gluconeogenesis, without altering ureagenesis despite reduced plane of nutrition. These homeorhetic adaptations are employed to reduce endogenous heat production while diverting amino acids to the growing fetus. Metabolic adaptation to heat stress in early lactation involves increased long-chain fatty acid degradation in muscle peroxisomes, allowance for muscle glucose utilization but diminished hepatic use of amino acid-derived pyruvate for gluconeogenesis and reduced peroxisomal fatty acid oxidation and ATP production in liver of HS compared to PF cows in early lactation. Consequently, metabolic adaptation to heat stress and reduced feed intake differ between late pregnancy and early lactation of dairy cows to maintain energy supply for fetus development or milk production simultaneously reducing endogenous heat production.

## Introduction

Worldwide 1.5 billion cattle (FAO, 2013) are negatively affected by extreme temperature changes with more frequent heat waves during summer periods [1]. Heat stress leads to decreased milk production, reduced reproduction rate and growth of dairy cows [2]. The economic losses caused by thermal stress was predicted to cost the US $879 million annually [3], Australia 6,838–11,986 $/per year for the cattle herd [4] and milk production in Europe is expected to drop by 5.7–7% [5,6]. Extended cooling costs and feeding systems for dairy cows [6] conflict with the growing demand on milk and beef production [7].

Metabolic heat production of dairy cows increases with the level of milk synthesis, making high-yielding dairy cows extremely susceptible towards environmental heat, whereas non-lactating cows being e.g. in the late pregnant period, produce less metabolic heat [8] and are less susceptible to environmental heat [9]. When ambient temperatures exceed the thermoneutral zone of lactating dairy cows, feed intake declines which contributes to reduction in milk production and loss of body weight [2,10]. Also, exposure of non-lactating, late-gestating cows to heat adversely affects milk yield, milk constitute contents, metabolic health [11], embryonic development [12], and trigger carry-over effects in the subsequent lactation [13]. Besides the reduction in energy expenditure, metabolic adaptation to thermal stress comprises also a shift in post-absorptive metabolism and nutrient partitioning aligned to reduce endogenous heat production [14,15]. It has been proposed that lack of adipose tissue mobilization during heat stress reduces metabolic heat production from fatty acid oxidation and that metabolic fuel selection shifts towards glucose utilization in lactating dairy cows [8,14,15]. We have recently shown using indirect calorimetry that whole body fat oxidation is blunted in cows experiencing heat stress, while fat oxidation increased in pair-fed cows at thermoneutrality [16]. Metabolic responsiveness towards high ambient temperatures, however, depends on the physiological stage. In contrast to lactating cows, adipose tissue of non-lactating, late pregnant cows is not refractory to lipolytic, adrenergic stimuli, and the rate of amino acid degradation was lower than in the postpartal stage [16]. However, the molecular mechanisms underlying the different adaptation processes during lactation and late pregnancy have not been thoroughly evaluated yet.

The liver is the main organ involved in glucose homeostasis by producing glucose from ruminal propionate [17]. During early lactation, gluconeogenesis and glycogenolysis are typically increased to provide glucose for milk lactose production [18,19]. The increase in gluconeogenesis is much more pronounced in cows calving during a hot summer as compared to a cold spring period [20] and presumably involves increased utilization of lactate and alanine for glucose synthesis [20,21]. Furthermore, hepatic mRNA expression of genes encoding fatty acid oxidation increases during the transition period from late pregnancy into lactation [18,19,22]. When cows, however, experience heat during the transition period, expression of genes associated with fatty acid oxidation is downregulated compared to control counterparts [20].

A second major source of stored glycogen but also amino acids is the skeletal muscle. Heat stress seems to have global effect on the amino acid metabolism resulting in an increased mobilization from skeletal muscle protein [23–25], but the molecular mechanisms underlying muscle proteolysis have not been investigated yet. As the muscle has a reduced capacity to oxidize fatty acids it presumably relies on circulating and glycogen-stored glucose for energy needs [25]. Regulation of pyruvate entry to the TCA cycle seems to play a major role to facilitates lactate and pyruvate-alanine flux to hepatic gluconeogenesis [25,26], although its contribution to cellular and system energetic homeostasis is unclear [27]. Therefore, the main objective of the current study was to evaluate molecular adaption of major catabolic and anabolic pathways in late pregnant and early lactation dairy cows to thermal stress conditions.

## Materials and Methods

### Animal experiments

All procedures were approved by the ethics committee of the State Government in Mecklenburg-West Pomerania, Germany (LALLF M-V/TSD/7221.3–1.1-074/12). As described earlier [16], 14 German Holstein cows genotyped for HDP70.1 5´UTR 895 [28] were grouped to heat-stressed (HS, n = 7) or pair-feeding (PF, n = 7) group. All cows were at the end of the 2^nd^ parity (ante partum, ap) and not milked within the 7 weeks prior to the expected calving date. Animals received a total mixed ration twice daily (at 0700 h and 1500 h) [16]. Both groups passed through a 13-day trial once in ante partum (HSap and PFap) and post-partum stage (HSpp and PFpp). Animals were halter-trained and well adapted to climate chambers with a light cycle ranging from 0600 to 1900 has described previously [16]. Three weeks before and after parturition, the 13-day trial consisted of two 6 days periods P1 and P2 separated by one day of thermal transition. In the experimental period P1, both HS and PF groups were exposed to the same climate conditions (15°C, 63±1% relative humidity (RH) resulting in a temperature-humidity-index (THI) of 60) with *ad libitum* feeding. On the following transition day, the air temperature was continuously increased to permanent 28°C for HS, but remained at 15°C for PF animals (experimental period P2). RH adjusted within 24 h to 52±2% for HS animals (THI = 76). THI was calculated as described earlier [16]. Feed intake was recorded daily. On the transition day and the following 6 days of period P2, reduction of daily ad libitum intake of HS cows was calculated as percentage of the mean daily intake in P1 to provide the same amount of feed to PF cows during P2 [15]. Cows had free access to water, which was tempered to 28°C for HS animals during P2. In the pp period, cows were milked at 0630 h and 1630 h and milk yield was determined daily [16]. Due to severe sickness of individual cows, which were withdrawn from trial, groups amounted to: HSap n = 7, PFap n = 6, HSpp n = 6, PFpp n = 6.

### Blood sampling and Analyses

At the first day of P1, cows were equipped with an indwelling jugular catheter (Certofix mono; B.Braun, Melsungen, Germany). Before morning feeding, daily blood samples were collected during period P1 and P2 into 9 ml-monovettes (Sarstedt, Nümbrecht, Germany) containing EDTA. Blood samples were centrifuged immediately after collection at 1,570 x g for 20 min at 4°C to obtain plasma which was stored at -80°C before analysis. Plasma insulin concentrations were determined by RIA (#1257; DRG Diagnostics, Marburg, Germany). Intra-assay variation was 3.7% and inter-assay variation 5.0–6.0%. Glucagon was detected by RIA (GL-32K; Linco Research, St. Charles, MO, USA) with an intra assay variation of 3.4% and an inter assay variation of 16.3–28.6%. Acyl ghrelin was measured as described previously [29] using a RIA kit (GHRA-88HK; Linco Research, St Charles, Billerica, MO, USA). The intra-assay variation was 4.0% and the inter-assay variation 6.8–16.6%. Plasma urea, albumin, alanine aminotransferase (ALT), β-hydroxybutyric acid (BHBA) and lactate were analyzed photometrically by ABX Pentra 400 (Horiba Medical, Kyoto, Japan) using the following kits (urea LT-UR0010, Labor+Technik Lehmann, Berlin, Germany; albumin A11A01664, ALT A11A01627; BHBA A11A01667; lactate A11A011721, Axonlab, Stuttgart, Germany). Plasma amino acids concentrations were analyzed by high-performance liquid chromatography as described recently [30].

### Biopsies and RT-RCR

*Semitendinosius* muscle and liver biopsies were taken before morning feeding on transition day (representing period P1) and on day 6 after PF or HS challenge (representing period P2), both ap and pp. Tissues were snap frozen in liquid nitrogen and stored at -80°C until analysis. Tissues were mortared under liquid nitrogen and total RNA was extracted from 50 mg tissue powder with TriFast Reagent (Peqlab, Erlangen, Germany). RNA isolation was performed by using an RNeasy kit (Qiagen, Valencia, CA) and RNA quality was assessed using an Agilent 2100 Bioanalyzer, yielding RIN factors for muscle and liver between 5.0 and 7.9 (median 6.95) and between 6.0 and 7.8 (median 7.3), respectively.

First strand cDNA synthesis (750 ng total RNA) was completed using 2400 U RevertAid Reverse Transcriptase (Thermo Fisher Scientific, Dreieich, Germany) and 250 pmol random primers (Metabion International, Planegg/Steinkirchen, Germany). cDNA was purified with High Pure PCR Product Purification Kit (Roche, Basel, Switzerland) and stored at -80°C until use. Transcriptional expression was quantified by real-time PCR. Primers were designed using Primer3 Plus software [31] or PrimerBLAST [32] (S1 Table). For liver, one PCR reaction contained 2 μl diluted cDNA (10 ng/μl), 5 μl H_2_O PCR grade, 400 nM of each primer, and 2 μl Light Cycler FAST DNA Master PLUS SYBR Green I Reaction Mix (Roche, Basel, Switzerland) and was carried out in duplicates using LightCycler 2.0 (Roche, Basel, Switzerland). For muscle tissue, qPCR reactions contained 2 μl diluted cDNA (10 ng/μl), 2 μl H2O PCR grade, 5 μl Luminaris Color HiGreen qPCR Master Mix (Roche, Basel, Switzerland) and 400 nM of each primer. Amplicons were sequenced on an ABI 3130 Genetic Analyzer (Life Technologies GmbH, Darmstadt, Germany) for quality control purpose. The obtained sequences were blasted using NCBI BLAST tool to confirm sequence identity. The efficiency of amplification was calculated using LinRegPCR software, version 2014.4 (Academic Medical Centre, Amsterdam, Netherlands; [33], yielding efficiency values between 1.84 and 1.91 (S1 Table). Data were quantified by qbasePlus software (Biogazelle, Gent, Belgium). The gene expression stability of five candidate reference genes was determined in geNorm. For final analysis were used *lipoprotein receptor-related protein 10* (LRP10) and *hippocalcin-like1* (HPCAL1) for liver and LPR10, *ceroid-lipofuscinosis neuronal 3* (CLN3) and *emerin* (EMD) for muscle tissue as references genes.

### Western Blot

Muscle tissue (50 mg) was homogenized in lysis buffer containing 50 mM Tris-HCl (pH 7.8), 1 mM EDTA, 10 mM NaF, 1% IGPEAL CA-630, 0.1% Triton X100, 0.5% deoxycholic acid (DCA) and 0.1% SDS. Protein concentrations were measured using Bradford method. Equal amounts of protein (50 μg) were separated by SDS-PAGE and transferred to a nitrocellulose membrane (Whatman Protran BA 83, Dassel, Germany). Membranes were blocked with 3% bovine serum albumin (BSA) and milk powder in Tris-buffered saline (TBS) containing 0.1% Tween 20 (TBST) for 1 h and then incubated overnight with the following primary antibodies: phosphorylated AMP-activated protein kinase Thr172 (pAMPK; #2535, Cell Signaling Technology, Cambridge, UK), AMPK (#2603, Cell Signaling Technology, Cambridge, UK), short/branched chain acyl-CoA dehydrogenase (ACADSB; LS-C81878/19265, Lifespan Bioscience Seattle, WA, USA) and glyceraldehyde-3-phosphate dehydrogenase (GAPDH; PA1-988, Thermo Scientific, Waltham, MA, USA). After incubation, membranes were washed in TBST, incubated for 1 h at room temperature with the corresponding secondary antibody (goat anti rabbit IgG HRP, sc-2004, Santa Cruz Biotechnology, Dallas, USA or donkey anti sheep IgG HRP, ab97125, Abcam Cambridge, USA) and washed again in TBST. Chemiluminescent reagents were applied and blots were exposed to hyperfilms (GE healthcare, Chalfont St Giles, Buckinghamshire, UK). Hyperfilms were scanned and quantified using ImageJ (version 1.49). The ratio of pAMPK/AMPK and ACADSB/GAPDH, respectively, were calculated.

### Statistical Analysis

Repeated measures data were analyzed for the effects of group, day, and their interaction during period 2 as a completely randomized design using PROC MIXED with repeated measurements analysis with day as the repeated effect. Data were tested for parametric or non-parametric distribution. For the comparison of group differences on a daily basis, a Tukey-Kramer test was applied. For PCR and Western Blot analyses, differences between periods P1 and P2 in the same group were analyzed using the Wilcoxon signed rank sum test included in the UNIVARIATE procedure of SAS (Version 9.4, SAS Institute Inc., Cary, NC, USA). Analysis of differences between HS and PF in P2 at the same productive stage was performed using the exact Wilcoxon-Mann-Whitney test of the NPAR1WAY procedure. We did not test for difference between ap and pp, thereby ignoring carry over effects. Repeated measurements are given as mean ± standard error (SEM). Results were considered as statistical significant at P<0.05 and trends between 0.05<P<0.07.

## Results

### Environmental heat downregulated PDK2 mRNA in skeletal muscle

**Fig 1:**
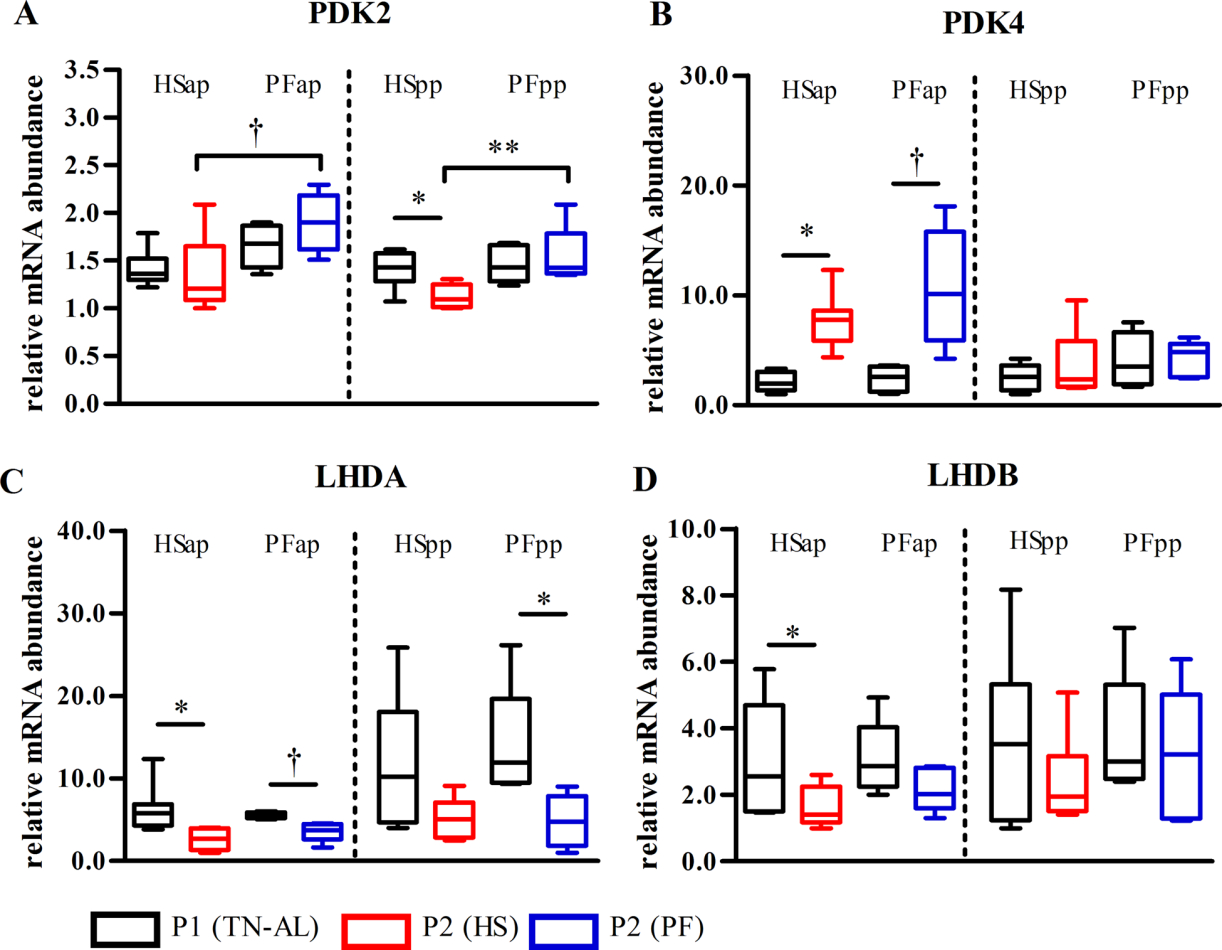
Regulation of PDC inhibitors and lactate dehydrogenase mRNA in skeletal muscle after heat stress or pair-feeding. mRNA abundances of (A) pyruvate dehydrogenase kinase isozyme 2 (*PDK2*), (B) isozyme 4 (*PDK4*), (C) lactate dehydrogenase A (*LDHA*) and (D) lactate dehydrogenase B (*LHDB*) in skeletal muscle of heat-stressed (HS) or pair-fed (PF) cows. Muscle samples were obtained after ad libitum feeding in P1at thermoneutral conditions (P1 (TN-AL), black) and after 6 days (P2) of HS (red) or PF (blue) cows in the ante partum (ap) and post partum (pp) period, respectively. Data are from HSap n = 7, PFap n = 5, HSpp n = 6, PFpp n = 6 RNA samples. ** P<0.01, * P<0.05, † 0.05< P< 0.07.

**Fig 2:**
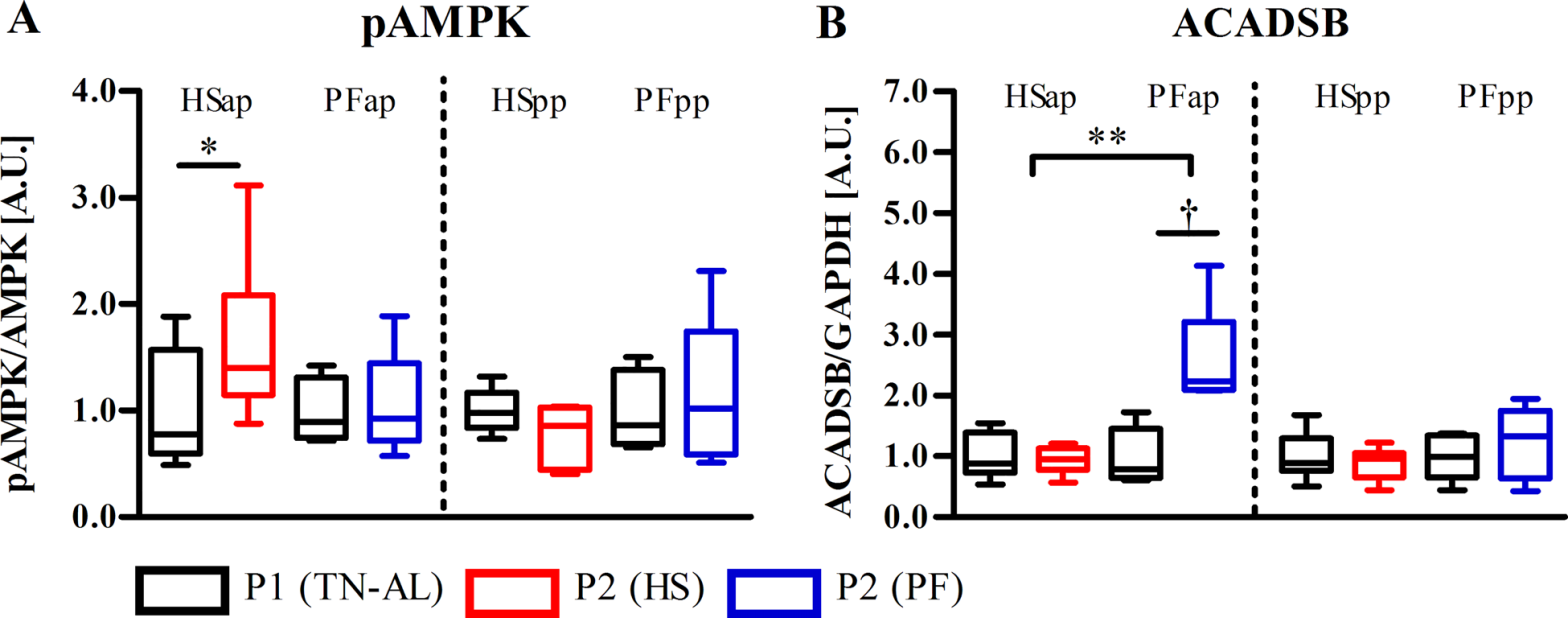
Thermal stress activated AMPK but prevented increase in muscle ACADSB in late gestation. (A) Phosphorypation of AMP-activated protein kinase (pAMPK) and (B) short/branched chain acyl-CoA dehydrogenase (ACADSB) in skeletal muscle of heat-stressed (HS) and or pair fed (PF) cows. Muscle samples for Western Blot were obtained after ad libitum feeding in P1 at thermoneutral conditions (P1 (TN-AL), black) and after 6 days (P2) of HS (red) or PF (blue) cows in the ante partum (ap) and post partum (pp) period, respectively. pAMPK was normalized to AMPK, whereas ACADSB was normalized to glyceraldehyde 3-phosphate dehydrogenase (GAPDH). Data are from HSap n = 6, PFap n = 5, HSpp n = 6, PFpp n = 5 RNA samples. ** P<0.01, * P<0.05, † 0.05< P< 0.07.

After heat exposure, expression of pyruvate dehydrogenase kinase isozyme 2 (*PDK2*) mRNA tended to be reduced (P<0.063) in late gestation and was significant decreased (P<0.01, Fig 1A) in early lactation in comparison to PF. Lactate dehydrogenase A and B (*LDHA*, *LDHB*) and *PDK4* remained unaltered between HS and PF in both stages (Fig 1B–1D). However, *PDK4* mRNA increased from P1 to P2 in late-gestating cows after exposure to HS (P<0.05), whereas *PDK2* mRNA did not (Fig 1A and 1B). In the pp period, only *PDK2*, but not *PDK4* mRNA expression decreased in HSpp, but not PFpp cows (P<0.05). A significant reduction of *LDHA* and *LDHB* mRNA expression was observed in late gestation, but not in early lactation of HS cows (Fig 1C and 1D). Furthermore, *LDHA* mRNA expression was significant reduced in PFpp cows (P<0.05; Fig 1C). Activation (phosphorylation) of AMP-activated protein kinase (AMPK) did not show differences between HS and PF, but comparing P1 to P2 showed an increased phosphorylation in HSap cows (P<0.05, Fig 2A).

### Heat stress prevented pair-feeding induced increased transcription regulating fatty acid oxidation in skeletal muscle

**Fig 3:**
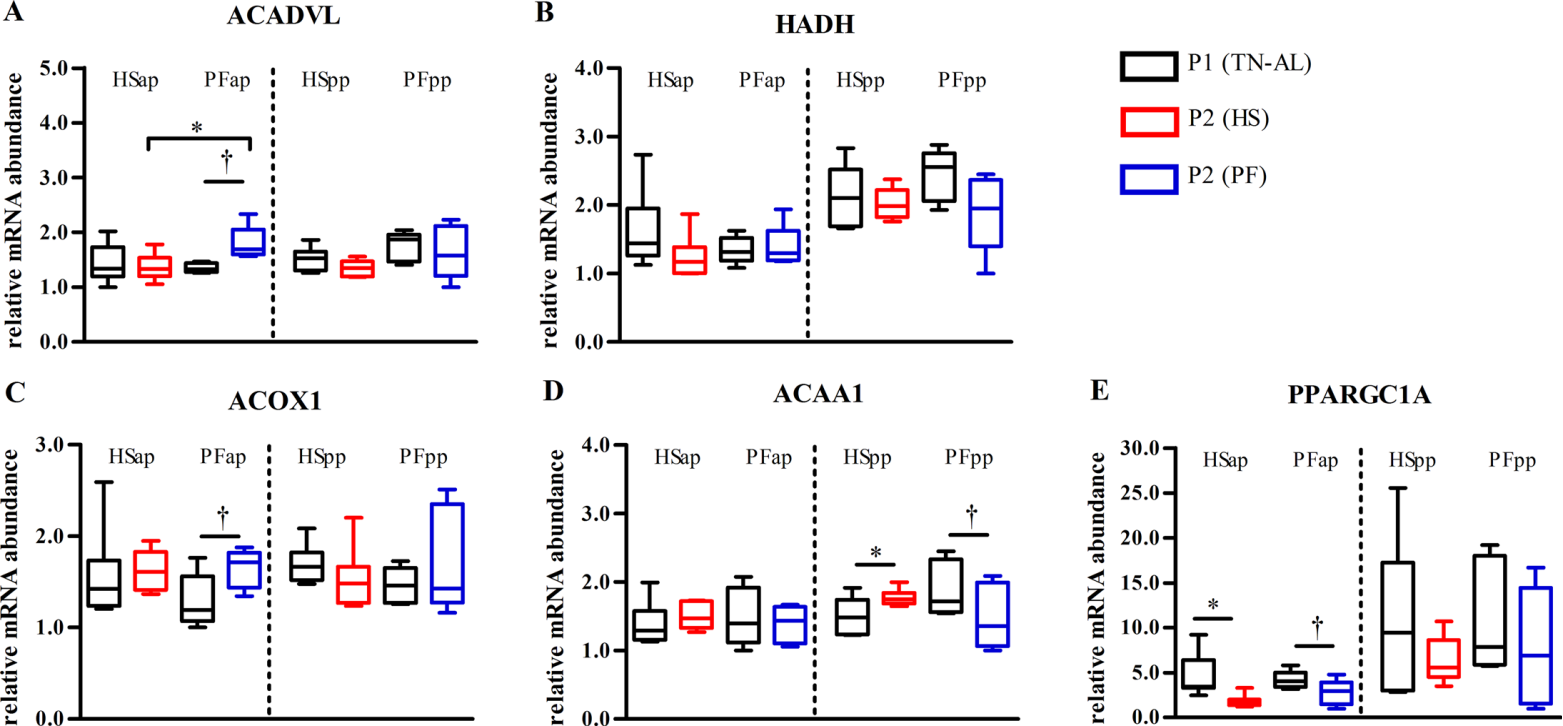
Muscle fatty acid oxidation gene expression is affected by heat stress. mRNA abundances of (A) acyl-CoA dehydrogenase very long chain (*ACADVL*), (B) 3-hydroxyacyl-CoA dehydrogenase (*HADH*), (C) peroxisomal acyl-coenzyme A oxidase 1 (*ACOX1)*, (D) peroxisomal acetyl-coenzyme A acyltransferase 1 (*ACAA1*), and (E) peroxisome proliferator-activated receptor gamma coactivator 1-alpha (*PPARGC1A*). Muscle samples were obtained after ad libitum feeding in P1 at thermoneutral conditions (P1 (TN-AL), black) and after 6 days (P2) of HS (red) or PF (blue) cows in the ante partum (ap) and post partum (pp) period, respectively. Data are from HSap n = 7, PFap n = 5, HSpp n = 6, PFpp n = 6 RNA samples. * P<0.05, † 0.05< P< 0.07.

While mRNA expression of mitochondrial acyl-CoA dehydrogenase, very long chain (*ACADVL*) tended to increase after PF (P<0.063), cows exposed to HS had significantly reduced *ACADVL* expression as compared to late gestation PF cows (P<0.05, Fig 3A). This effect could not be observed during early lactation. Also, protein expression of short/branched chain acyl-CoA dehydrogenase (ACADSB) was significantly lower in HS compared to PF cows during late gestation (P<0.01, Fig 2B), while it was unaffected in early lactation.

However, mRNA abundances of mitochondrial 3-hydroxyacyl-CoA dehydrogenase *(HADH*) peroxisomal acyl-coenzyme A oxidase 1 (*ACOX1*), acetyl-coenzyme A acyltransferase 1 (*ACAA1*) and remained unaltered in late-gestating HS cows (Fig 3B–3D). In early lactation, HS cows showed a significant increase in mRNA expression of the fatty acid activating enzyme *ACAA1* (P<0.05, Fig 3D), while mRNA abundances of the oxidative enzymes *ACADVL*, *ACOX1* and *HADH* were not affected in early lactation HS or PF cows (Fig 3A, 3B and 3D). The mRNA expression of peroxisome proliferator-activated receptor gamma coactivator 1-alpha (*PPARGC1A*), a master regulator of mitochondrial biogenesis, declined from P1 to P2 in late-gestating HS cows (P<0.05, Fig 3E), whereas in early lactation mRNA abundance did not change.

### Heat stress induced mRNA expression of FOXO3 controlling proteolysis in skeletal muscle

**Fig 4:**
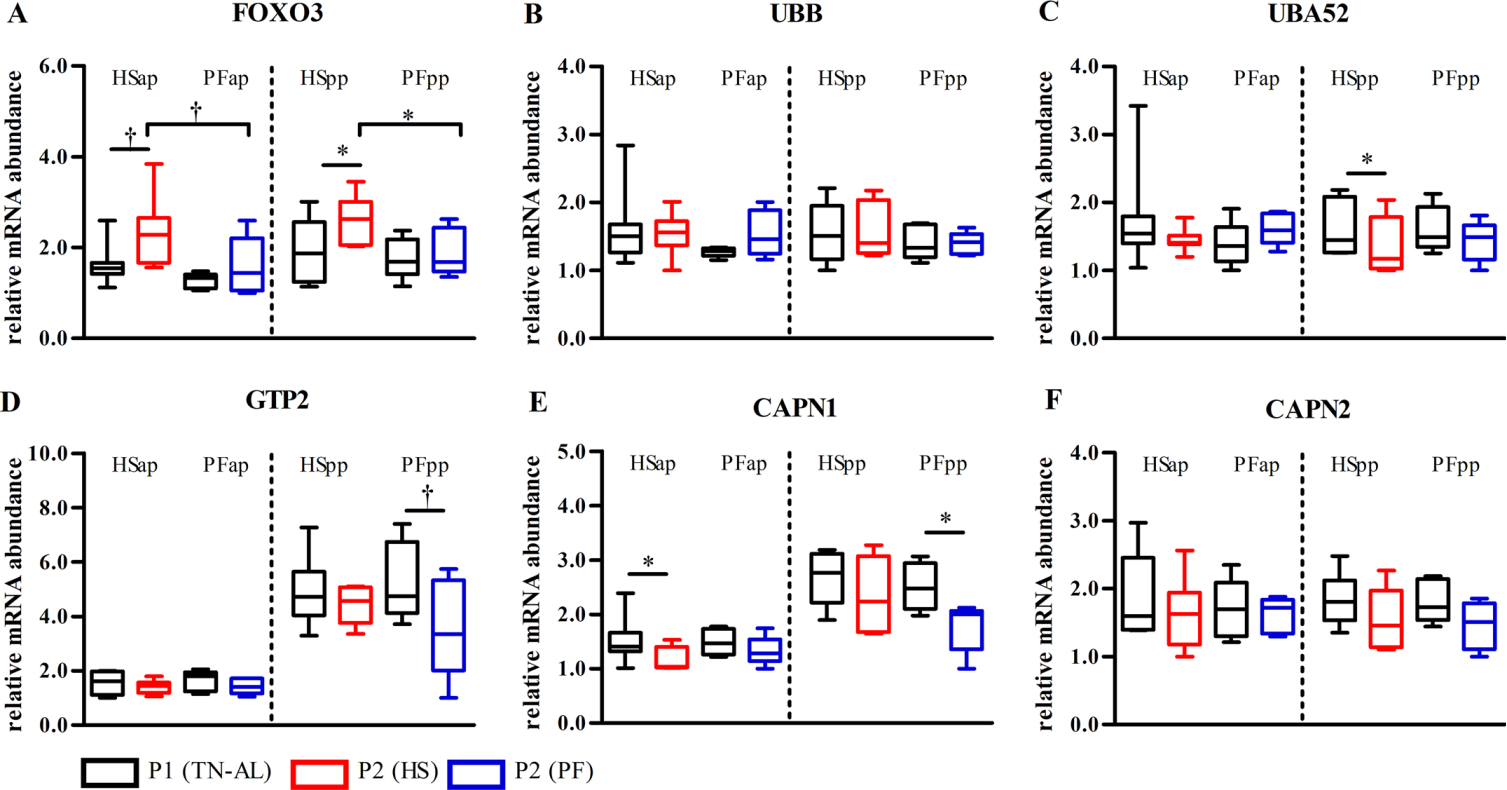
FOXO3 mRNA expression is upregulated by thermal stress but not after pair-feeding in skeletal muscle. Muscle samples were obtained after ad libitum feeding in P1 at thermoneutral conditions (P1 (TN-AL), black) and after 6 days (P2) of HS (red) or PF (blue) cows in the ante partum (ap) and post partum (pp) period, respectively. mRNA abundances of (A) forkhead box O3 (*FOXO3*), (B) ubiquitin B (*UBB*), (C) ubiquitin A-52 ribosomal protein fusion product 1 (*UBA52*), (D) alanine aminotransferase 2 (*GPT2*), (E) calpain 1 (*CAPN1*), (F) calpain 2 (*CAPN2*). Data are from HSap n = 7, PFap n = 5, HSpp n = 6, PFpp n = 6 RNA samples. * P<0.05, † 0.05< P< 0.07.

The mRNA abundance of forkhead box O3 (*FOXO3*), a transcriptional regulator of protein degradation tended to be elevated in late-gestating HS (P<0.07) and was significantly upregulated in early lactation HS cows (P<0.05, Fig 4A). Pair-feeding did not affect *FOXO3* abundances (Fig 4A), resulting in significantly higher FOXO3 expression in HS compared to PF cows in both late pregnancy and early lactation (Fig 4A). The mRNA expressions of the proteolytic enzymes alanine aminotransferase 2 (*GPT2*) and calpain 1 (*CAPN1*) decreased after PF but was not affected by HS during early lactation (Fig 4D and 4E), however, differences between HSpp and PFpp groups did not reach significance. *CAPN1* mRNA decreased from P1 to P2 in HSap and PFpp cows (P<0.05, Fig 4E) and *UBA52* mRNA expression declined after HS exposure in the pp stage (P<0.05, Fig 4C). However, mRNA expressions of the proteolytic enzymes ubiquitin B (*UBB*), and calpain 2 (*CAPN2*) were not affected by HS or PF conditions (Fig 4B and 4F).

### Heat stress influenced hepatic PCK1 and PC mRNA expression

**Fig 5:**
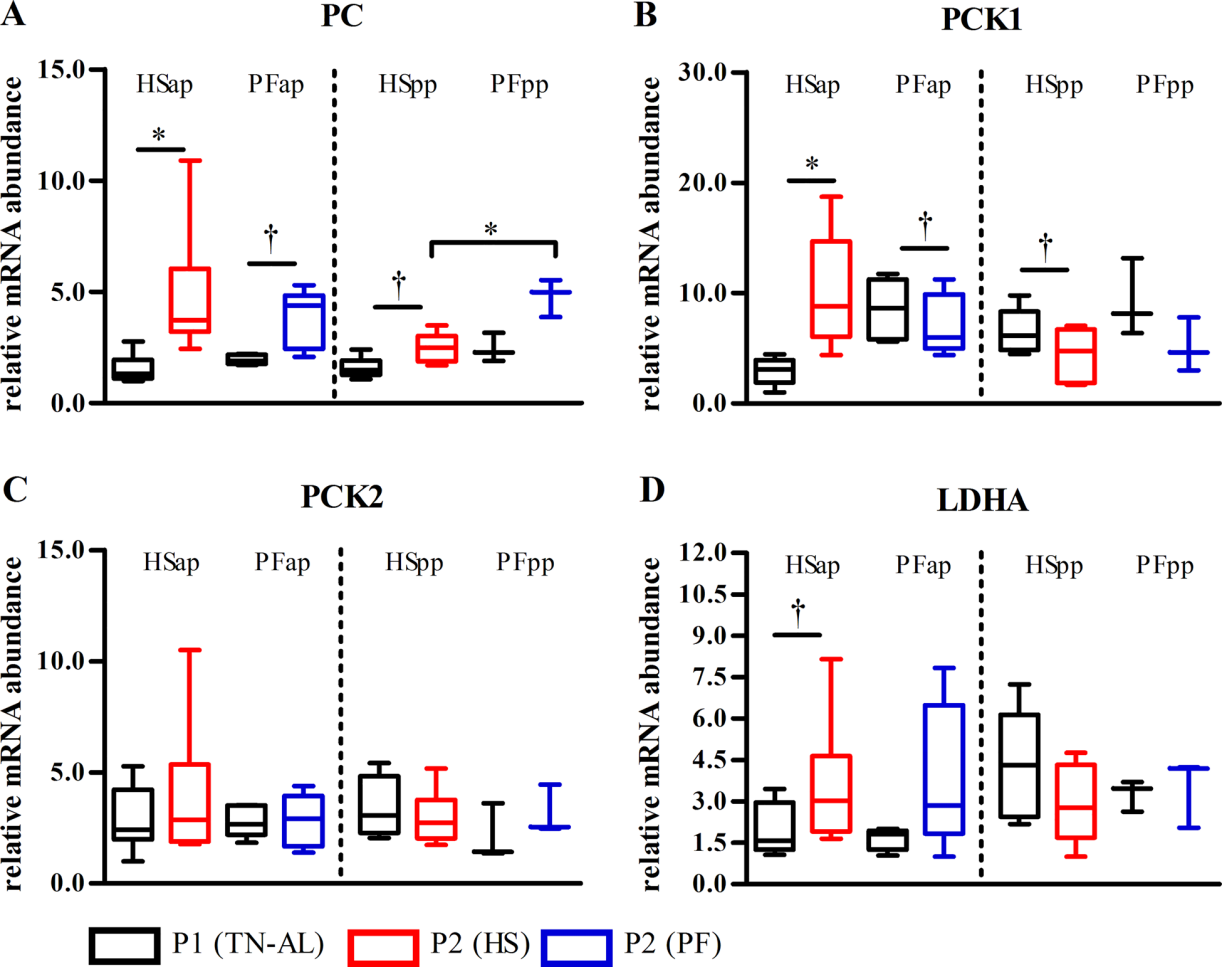
Heat stress effects on hepatic gluconeogenic pathway mRNA expression. mRNA abundances of (A) pyruvate carboxylase (*PC*), (B) phosphoenolpyruvate carboxykinase 1 (*PCK1*), (C) phosphoenolpyruvate carboxykinase 2 (*PCK2*), (D) lactate dehydrogenase A (*LDHA*) in liver of heat-stressed (HS) and or pair-fed (PF) cows. Liver samples were obtained after ad libitum feeding in P1 at thermoneutral condition (P1 (TN-AL), black) and after 6 days (P2) of HS (red) or PF (blue) cows in the ante partum (ap) and post partum (pp) period, respectively. Data are from HSap n = 6, PFap n = 5, HSpp n = 6, PFpp n = 3 RNA samples. * P<0.05, † 0.05< P< 0.07.

Pyruvate carboxylase (*PC*) mRNA expression increased or tended to increase from P1 to P2 in HS and PF cows (Fig 5A), but only in early lactation PC abundance was significantly lower under HS compared to PF conditions (P<0.05). The expression of cytosolic phosphoenolpyruvate carboxykinase 1 (*PCK1*) increased from P1 to P2 in HS but tended to decrease in PF cows before parturition, however, there was no significant difference between HSap and PFap cows (Fig 5B). In early lactation, PCK1 abundance tended to decrease after HS (P<0.063) but not after PF, without reaching significant group differences (Fig 5B). Mitochondrial phosphoenolpyruvate carboxykinase 2 (*PCK2*) did not differ between HS and PF in both stages (Fig 5C). Furthermore, *LDHA* mRNA expression tended to increase in P2 relative to P1 in HS in late gestation (P<0.063, Fig 5D), whereas in early lactation no changes could be detected.

### Heat stress did not alter mRNA expression of peroxisomal and mitochondrial fatty acid oxidation in liver

**Fig 6:**
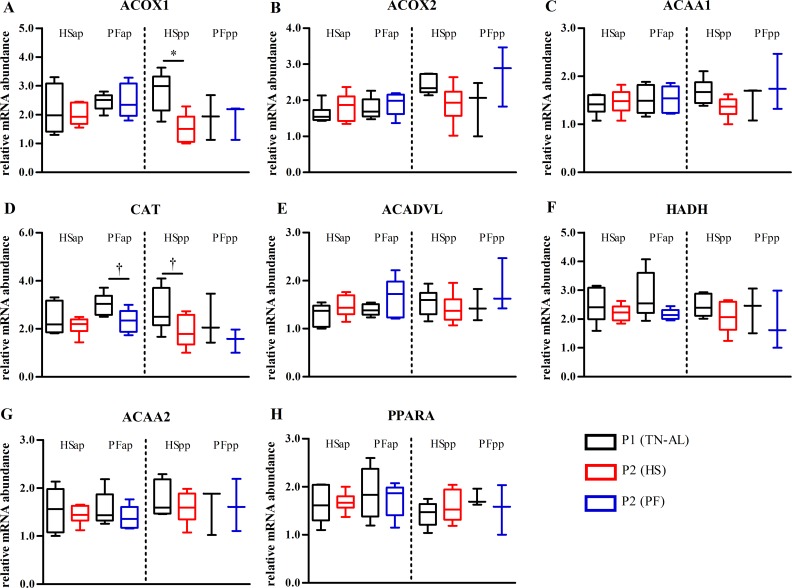
Unchanged mRNA expression of hepatic fatty acid oxidation in heat-stressed cows. mRNA abundances of (A) acyl-CoA oxidase 1 (*ACOX1*), (B) acyl-CoA oxidase 2 (*ACOX2*), (C) peroxisomal acyl-coenzyme A oxidase 1 (*ACAA1*), (D) catalase (*CAT*), (E) acyl-CoA dehydrogenase very long chain (*ACADVL*), (F) 3-hydroxyacyl-CoA dehydrogenase (*HADH*), (G) 3-ketoacyl-CoA thiolase (*ACAA2*), (H) peroxisome proliferator-activated receptor alpha (*PPARA*) in liver of heat-stressed (HS) and or pair-fed (PF) cows. Liver samples were obtained after ad libitum feeding in P1 at thermoneutral condition (P1 (TN-AL), black) and after 6 days (P2) of HS (red) or PF (blue) cows in the ante partum (ap) and post partum (pp) period, respectively. Data are from HSap n = 6, PFap n = 5, HSpp n = 6, PFpp n = 3 RNA samples. * P<0.05, † 0.05< P< 0.07.

Comparison between HS and PF did not reveal differences in peroxisomal *ACOX1*, acyl-CoA oxidase 2 (*ACOX2*), *ACAA1*, catalase (*CAT*), *ACADVL*, *HADH*, 3-ketoacyl-CoA thiolase (*ACAA2*) and peroxisome proliferator-activated receptor alpha (*PPARA*) mRNA abundances in late-gestating and early lactation animals (Fig 6A–6H). By contrast, early lactation HS but not PF cows showed a reduction in peroxisomal *ACOX1* (P<0.05) and *CAT* (P<0.063) from P1 to P2 (Fig 6A and 6D), whereas CAT abundance also tended to decrease in late pregnancy after PF (P<0.063, Fig 6D).

### No transcriptional adaption of the urea and TCA cycle in liver during heat stress

**Fig 7:**
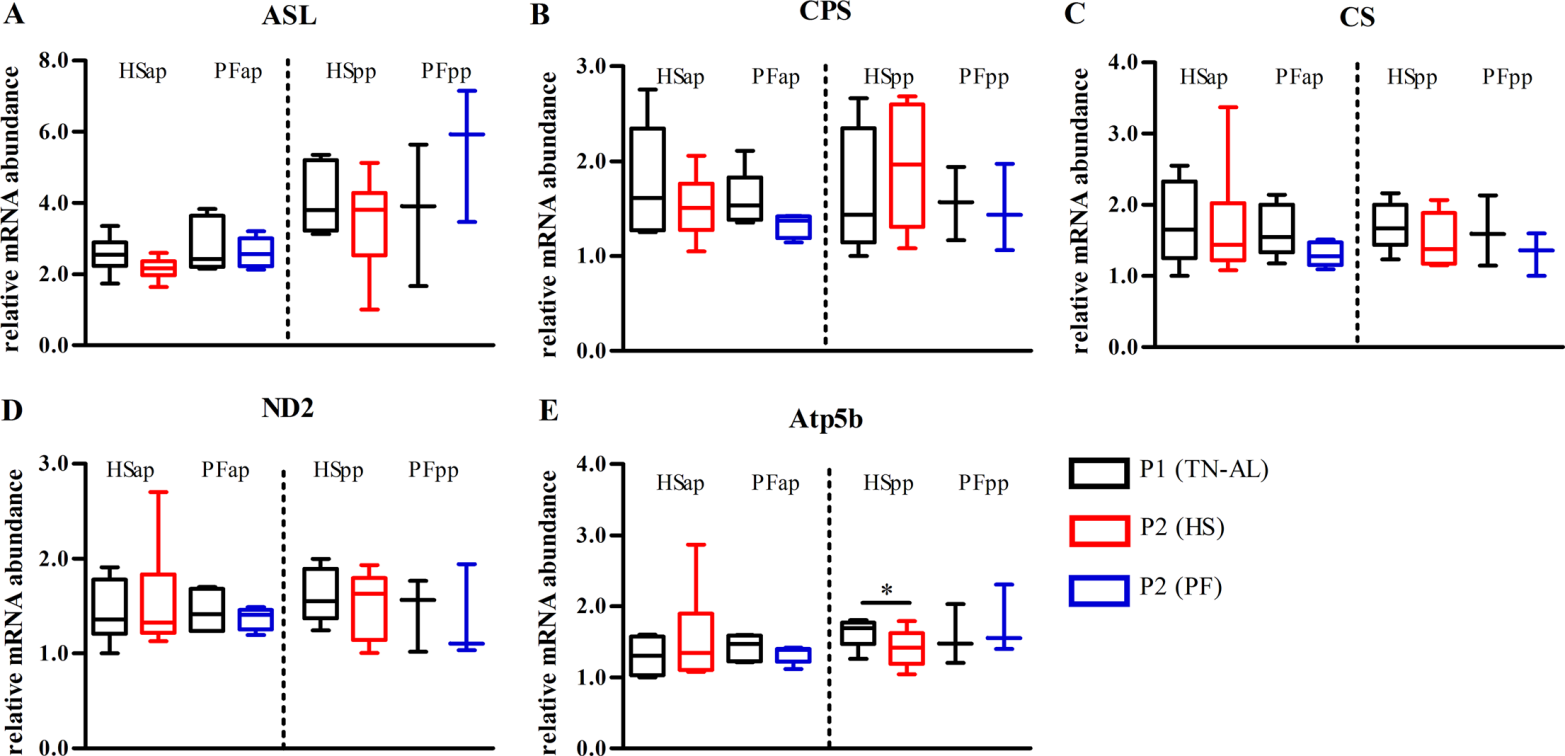
Unaltered gene expression of urea and TCA cycling, but decreased ATP5B mRNA in liver after thermal stress in early lactation. mRNA abundances of (A) cytosolic argininosuccinate lyase (*ASL*), (B) mitochondrial carbamoyl-phosphate synthase (*CPS*), (C) citrate synthase (*CS*), (D) NADH dehydrogenase subunit 2 (*ND2*), (E) mitochondrial F1 complex of ATP synthase, beta polypeptide (*ATP5B*) in liver of heat-stressed (HS) and or pair-fed (PF) cows. Liver samples were obtained after ad libitum feeding in P1 at thermoneutral condition (P1 (TN-AL), black) and after 6 days (P2) of HS (red) or PF (blue) cows in the ante partum (ap) and post partum (pp) period, respectively. Data are from HSap n = 6, PFap n = 5, HSpp n = 5, PFpp n = 3 RNA samples.* P<0.05.

HS and PF had no effect on the mRNA abundance of enzymes of the urea and the TCA cycle such as argininosuccinate lyase (*ASL*), mitochondrial carbamoyl-phosphate synthase (*CPS*) and citrate synthase (*CS*) in late gestation and early lactation (Fig 7A–7C). The mRNA expression of NADH dehydrogenase subunit 2 (*ND2*) and ATP synthase H^+^ transporting, mitochondrial F1 complex, beta polypeptide (*ATP5B*), did not differ between HS and PF (Fig 7D and 7E), but *Atp5b* mRNA abundance was lower in P2 compared to P1 in HSpp cows (P<0.05, Fig 7D), without reaching significant difference between groups.

### Heat stress increases plasma alanine in late gestation but decreased plasma glycine and proline in early lactation

Plasma alanine concentrations were 1.3-fold higher after HS relative to PF in late gestation (P<0.05), but did not differ in early lactation (Table 1). Glycine and proline concentrations were significantly lower whereas phenylalanine was higher in HS relative to PF cows in early lactation (P<0.05). Plasma amino acids involved in the urea cycle, namely citrulline, arginine and ornithine declined from P1 to P2 in HSap cows (P<0.031, Table 1). With the exception of arginine, these decreases could also be observed in PFap cows (P<0.031). Furthermore, glutamate, glutamine, asparagine, tyrosine, histidine, threonine, tyrosine, valine, methionine and lysine declined from P1 to P2 in PF cows during late gestation (all P<0.031), but not in early lactation. However, the sum of ketogenic amino acids were lower in P2 compared to P1 in HSap (P<0.031), whilst in PFap cows there was only a trend (P<0.063). Furthermore, threonine, tryptophan and leucine concentrations declined, whereas glycine concentration increased in HSap cows from P1 to P2 (P<0.031). In the early lactation period, significant reductions in plasma amino acid concentrations were only detected for glutamate and glycine plasma in HS cows (P<0.031).

**Table 1:** Plasma amino acids, urea, albumin and concentrations of heat-stressed and pair-fed cows on day 6 of each period P1 and P2. Period P1of thermoneutral conditions with ad libitum feeding; period P2 of either pair-feeding at thermoneutral conditions or during ad libitum feeding under heat exposure. HSap n = 7, PFap n = 5, HSpp n = 6, PFpp n = 6. Data are given as mean ± SEM.

	*ante partum*				*post partum*			
	heat stress		pair-fed		heat stress		pair-fed	
Metabolite	P1	P2	P1	P2	P1	P2	P1	P2
Asp	5.3 ± 1.7	4.9 ± 1.2	4.4 ± 0.6	5.5 ± 1.7	5.4 ± 2.1	4.8 ± 0.9	4.2 ± 0.9	3.3 ± 0.8
Glu	56.6 ± 6.2	48.6 ± 2.6	70.7 ± 8.6	44.5 ± 6.5+	42.2 ± 7.2	32.3 ± 3.8+	39.1 ± 1.7	27.4 ± 4.2#
Cys	36.2 ± 4.1	36.2 ± 4.3	31.1 ± 6.1	29.0 ± 5.1	31.4 ± 4.9	34.7 ± 5.5	23.8 ± 3.4	22.5 ± 4.0
Asn	34.2 ± 2.6	28.5 ± 2.1	33.4 ± 2.6	24.6 ± 2.3+	41.3 ± 5.0	31.3 ± 1.6	43.7 ± 2.9	33.9 ± 6.2
Ser	62.9 ± 3.3	57.2 ± 3.9	67.0 ± 5.2	55.5 ± 7.0	66.6 ± 11.7	35.8 ± 5.9	69.7 ± 8.4	46.0 ± 12.2
Gln	351.9 ± 13.3	360.8 ± 18.3	396.3 ± 23.3	331.9 ± 12.6#	271.5 ± 19.1	252.5 ± 19.4	277.2 ± 17.2	242.2 ± 23.1
His	59.5 ± 4.1	50.8 ± 3.9#	56.0 ± 3.5	45.4 ± 2.6+	60.7 ± 12.2	43.4 ± 2.9	41.5 ± 6.1	40.6 ± 5.5
Gly	224.6± 13.8	336.7 ± 18.9+	289.9 ± 20.7	393.1 ± 30.0#	504.4 ± 32.9	310.0 ± 31.8+	503.1 ± 45.3	436.7 ± 26.5*
Thr	79.9 ± 9.3	42.2 ± 3.1+	82.5 ± 10.2	48.4 ± 4.7+	65.1 ± 9.8	49.5 ± 4.5	68.0 ± 10.6	60.8 ± 10.8
Cit	76.9 ± 5.1	58.8 ± 5.1+	66.8 ± 4.8	53.2 ± 4.2+	82.3 ± 3.1	81.8 ± 9.0	91.5 ± 9.8	80.2 ± 9.9
Arg	82.9 ± 3.5	63.0 ± 5.1+	61.9 ± 5.2	69.6 ± 18.0	69.2 ± 7.2	74.2 ± 7.7	69.0 ± 2.5	61.9 ± 8.1
Ala	216.8 ± 18.0	220.3 ± 17.1	196.7 ± 18.5	168.1 ± 10.3*	191.0 ± 29.3	131.5 ± 19.6	183.9 ± 10.6	128.6 ± 32.4
Tyr	48.2 ± 9.7	29.0 ± 5.2	50.4 ± 3.3	34.5 ± 2.5+	45.0 ± 6.5	36.8 ± 2.5	47.0 ± 4.9	37.8 ± 7.8
Val	241.7 ± 46.2	152.8 ± 27.4 #	218.3 ± 14.8	147.6 ± 26.7+	244.5 ± 42.7	255.3 ± 52.2	262.5 ± 21.8	216.3 ± 39.8
Met	25.1 ± 1.2	20.6 ± 0.7	24.4 ± 2.1	20.0 ± 1.8	21.7 ± 2.4	18.3 ± 2.7#	17.6 ± 1.7	16.8 ± 2.4
Trp	45.0 ± 2.6	26.2 ± 2.2+	43.0 ± 2.2	23.2 ± 2.1+	39.0 ± 4.3	35.6 ± 2.0	40.4 ± 1.9	32.7 ± 5.9
Phe	62.9 ± 1.6	53.7 ± 4.9	49.8 ± 1.8	44.0 ± 3.7	47.6 ± 3.9	58.6 ± 2.1	50.3 ± 2.0	45.4 ± 4.7*
Ile	132.9 ± 8.5	99.4 ± 6.7	115.1 ± 9.5	102.4 ± 6.2	165.9 ± 9.5	182.2 ± 23.7	169.1 ± 24.1	149.1 ± 20.0
Orn	39.8 ± 5.0	22.6 ± 4.8+	39.4 ± 5.3	22.1 ± 2.9+	29.5 ± 5.0	31.0 ± 5.0	27.3 ± 2.1	26.3 ± 4.3
Leu	152.9 ± 9.4	119.6 ± 8.0+	119.6 ± 8.3	111.6 ± 8.2	167.7 ± 10.7	185.2 ± 14.3	143.6 ± 12.8	164.7 ± 21.5#
Lys	85.6 ± 13.2	69.7 ± 10.3	78.3 ± 6.8	61.7 ± 17.9+	93.5 ± 12.5	101.0 ± 12.3	70.5 ± 5.7	80.1 ± 10.1
Pro	21.2 ± 2.4	20.8 ± 2.8	20.8 ± 2.6	21.1 ± 7.3	22.1 ± 3.1	21.7 ± 2.2	24.2 ± 191	30.7 ± 2.9*
TotalAA	2244 ± 129	2038 ± 69	2197 ± 142	1945 ± 64	2437 ± 127	2128 ± 124	2387 ± 61	2109 ± 208
Ketogenic AA	238± 19	189± 17+	198± 15	173± 15#	261± 18	286± 25	214± 16	245± 30
Glucogenic AA	1871 ± 113	1724 ± 62	1864 ± 126	1677 ± 59	2042 ± 104	1740 ± 113	2020 ± 42	1748 ± 179
Urea [mmol/l]	3.8 ± 0.3	3.9 ± 0.4	2.7 ± 0.3	3.1 ± 2.4	3.6 ± 0.4	6.3 ± 1.3+	3.2 ± 0.1	3.6 ± 0.2#,*
Albumin [g/l]	30.9 ± 0.6	30.1 ± 0.8	30.2 ± 0.5	29.3 ± 0.4	33.4 ± 1.0	32.3 ± 0.8#	31.7 ± 0.5	32.2 ± 0.7
ALT [U/l]	21.3 ± 1.7	15.7 ± 0.9+	19.0 ± 1.5	13.8 ± 0.8+	20.3 ± 1.1	16.8 ± 0.4+	17.5 ± 1.5	16.2 ± 1.1
BHBA [mmol/l]	0.46 ± 0.3	0.67 ± 0.06+	0.49 ± 0.04	0.64 ± 0.1	1.3 ± 0.3	1.7 ± 0.7	1.04 ± 0.1	2.09 ± 0.4+
Ghrelin [ng/ml]	61.3 ± 12.4	72.8 ± 13.2	46.9 ± 10.1	111.8 ± 46.8	44.2 ± 5.0	44.8 ± 10.4	47.4 ± 9.1	75.6 ± 38.4

* P <0.05 for PF relative to HS (MWU test for group effects)

+ P <0.05 in P2 relative to P1, and

# indicates a trend (0.07<P<0.05) in P2 relative to P1 (Wilcoxon signed-rank test for paired samples)

### Increased plasma urea concentrations due to thermal stress in late gestation but not in early lactation

Plasma urea concentrations increased from P1 to P2 in early lactation HS cows and were significantly higher as compared to PF cows (P<0.05), whose concentrations only marginally increased (P<0.063; Table 1). On the other hand, plasma urea was unaffected in the ap period. Albumin, alanine amino transferase (ALT), and beta-hydroxybutyrate (BHBA) did not differ between HS and PF but plasma albumin tended to be lower in P2 compared to P1 in HSpp cows (P<0.063). Analysis of plasma BHBA revealed an increase from P1 to P2 in HSap cows (P<0.05), which was not observed in PF animals (Table 1). Furthermore, PFpp cows showed greater plasma BHBA concentrations in P2 (P<0.05, Table 1). In the ap period, plasma ALT concentrations declined from P1 to P2 in HS and PF (P<0.05). HSpp but not PFpp cows showed lower ALT concentrations in P2 relative to P1 (P<0.05).

### Decreasing insulin concentration results in the reduction of the insulin/glucagon ratio in HSap cows

**Fig 8:**
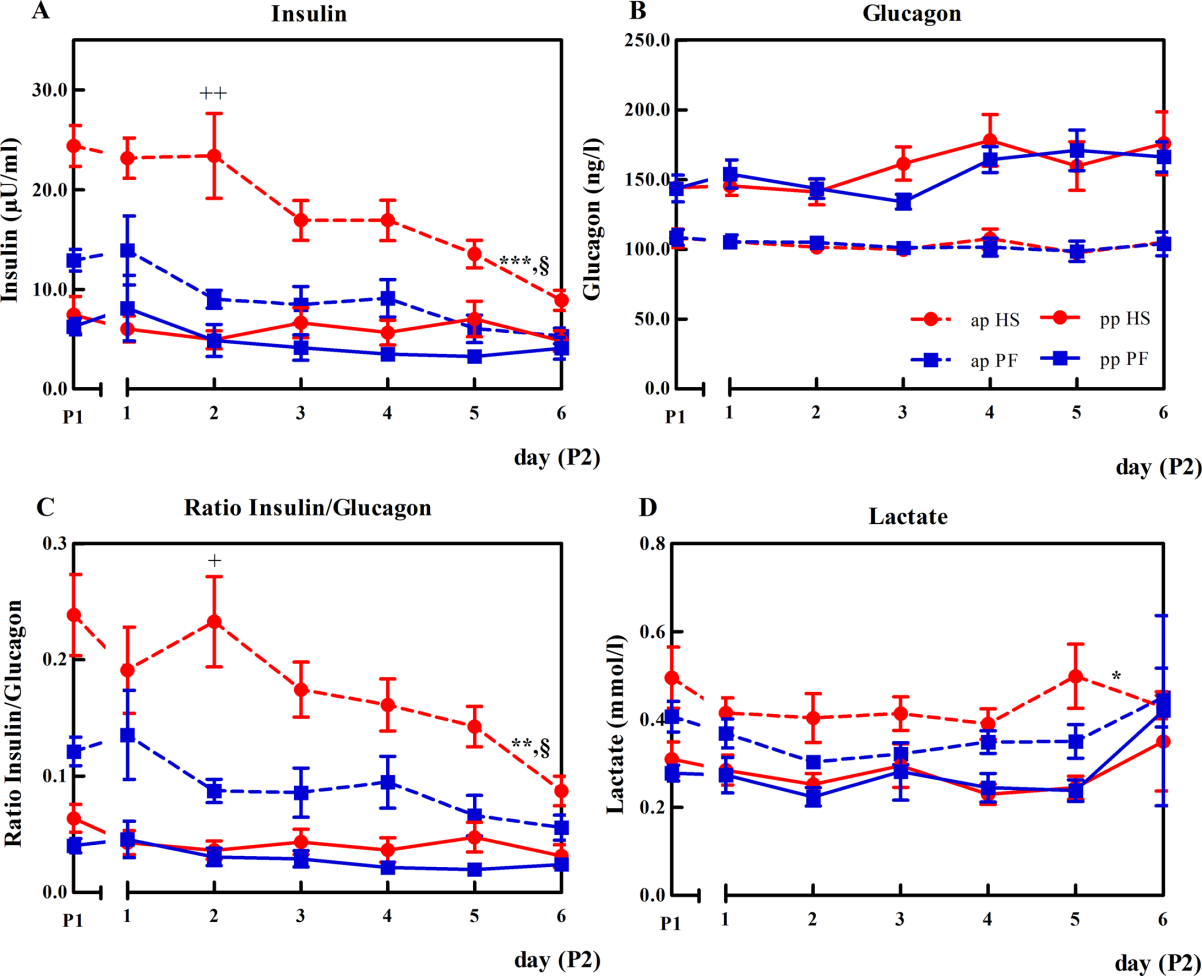
The effects of heat stress or pair-feeding on plasma insulin, glucagon and lactate. (A) plasma insulin in [μU/ml], (B) plasma glucagon [ng/l], (C) ratio insulin/glucagon, (D) plasma lactate [mmol/l]. In experimental period (P1) all animals were kept under thermoneutral conditions (THI = 60) with ad libitum feeding for six days. During period 2 (P2), HS cows (red) were heat-stressed (THI = 76), whereas PF cows (blue) were pair-fed in thermoneutrality (THI = 60) for six days in ante partum (ap, dashed lines) and again in post partum (pp, solid lines). The mean of the last days of P1 was calculated. Data are from HSap n = 6, PFap n = 6, HSpp n = 4, PFpp n = 6 and are presented as mean ± SEM. *** P<0.001, **P<0.01, *P<0.05 for group effects; § P<0.05 for time effects; ++ P<0.01 and + P<0.05 for HS vs. PF comparisons in the ap period (Tukey-Kramer test).

In late gestation, plasma insulin concentrations differed between HS and PF cows during P1, however, only in HS exposure led to a significant decrease during P2 (P<0.001, Fig 8A) while it remained constant in PF cows (ΔHS 15.4 vs. ΔPF 7.59 μU/ml; P<0.001). In early lactation, insulin concentrations did not change between groups in P1 or P2. Glucagon concentrations did also not differ between HS and PF cows during both stages (Fig 8B). The insulin/glucagon ratio declined in HSap cows during P2 (P<0.001, Fig 8C). Furthermore, HS cows showed higher lactate concentrations compared to PF cows in the ap (P = 0.05) but not in the pp period (Fig 8D). In addition, plasma ghrelin was not affected by HS or PF and remained unaltered in ante partum and pp stage (Table 1).

## Discussion

Periods with longer heat waves are predicted to occur more often leading to tremendous changes in animal farming in the next decades. Due to their enormous metabolic rate, but also due to negative energy balance during early lactation, dairy cows are particularly sensitive to heat during the transition period from pregnancy to lactation. The perspective of this study was to determine the effect of heat stress on metabolism and expression of key genes involved in energy metabolism of liver and muscle tissue of dairy cows during late gestation and early lactation.

### Heat stress in late gestation

Feed intake of late-gestating and early lactation cows was shown to decline by ~50% during heat periods [16,23]. Under the conditions of reduced energy intake, muscle metabolism enters a glucose-sparing state at thermoneutrality, while it appears to increase aerobic glycolysis during heat stress [2]. Muscle glucose metabolism is among others regulated by the activation of AMPK upon increase in cellular AMP/ATP ratio [34]. Muscle pAMPK is not significantly different between HS and PF cows in late gestation despite AMPK phosphorylation increased after one week of heat stress, but not after pair-feeding. This result indicates that heat stress might regulate intracellular energy balance via AMPK activation to shut down energy consuming anabolic processes and induce catabolic pathways [35].

The activity of the pyruvate dehydrogenase complex (PDC) is regulated by various PDKs while their individual functions are still not entirely resolved [36]. *PDK2* mRNA expression tended to be greater in PF compared to HS cows, indicating decreased PDC activity and reduced conversion of pyruvate to acetyl-CoA in PF cows. This regulation allows PF but not HS late-gestating cows to utilize acetyl-CoA deriving from fatty acid oxidation, an assumption supported by the greater muscle *ACADVL* mRNA and ACADSB protein abundance as well as greater whole-body fat oxidation in PF compared to HS cows [16]. Conclusively, HS cows limit heat production by preventing increase in fatty acid oxidation in skeletal muscle tissue.

Phosphorylation of AMPK signals to activate *FOXO3* gene expression [34]. Our data show that HS cows express more *FOXO3* mRNA compared to PF animals. FOXO3 is involved in the regulation of muscle proteolysis [37,38], which is reflected by a significant increase in plasma 1-/3-methylhistidine after heat exposure but not PF of late-gestating animals [16]. Although *FOXO3* mRNA expression increased under thermal stress, higher mRNA abundance of calpains (calcium- dependent, non-lysosomal proteases) and ubiquitin proteasome system could not be detected. However, lack of altered mRNA expression does not exclude the involvement of the lysosomal pathway degrading muscle proteins, but this assumption needs further investigation. Total plasma amino acid concentrations were not altered after HS or PF challenge, but interestingly, plasma alanine concentration did not decline after HS in pregnant cows as they do in PF counterparts. This result might indicate a continuously proceeding Cahill cycle during HS, presumably to meet the high amino acid requirement of the fast-growing near term-fetus. Activation of Cahill cycling requires sufficient glucose supply and the shut-down of pathways competing for the use of muscle pyruvate. Accordingly, we found reduced LDHB mRNA expression in HS but not in PF cows, indicating less conversion of pyruvate to lactate and back. Also, plasma lactate concentrations were greater in late gestating HS than PF cows, further pointing to an alleviation of Cori in favor of Cahill cycling.

Furthermore, hepatic PCK1 mRNA expression increased after HS but declined after PF, yet without reaching significance level between groups. Despite of this, opposed regulation of *PCK1* expression after HS and PF challenge may indicate that HS late-pregnant cows favor the cytosolic phosphoenolpyruvate (PEP) pathway, probably to export NADH to the cytosol necessary for gluconeogenesis. Favoring cytosolic over mitochondrial PEP production would allow greater mitochondrial NADH oxidation and thereby reducing mitochondrial heat production, an effect which does not occur in metabolically challenged early lactation cows. In addition, hepatic *PC* mRNA expression increased from P1 to P2 in both HS and PF animals and was not different between groups indicating that the level of feed intake but not heat stress *per see* regulates PC. In line with this, Shahzad et al. (2015) reported that greater *PC* mRNA abundance in transition cows with summer compared to winter calving and concluded that the expression responses could be related to lower DMI [20].

### Heat stress in early lactation

Homeorhetic adaptation to early lactation involves reduced glucose utilization and increased lactate efflux from skeletal muscle, thereby decoupling the Cori cycle to contribute to the sudden enormous glucose requirements of the mammary gland [39]. We found that HS in early lactation cows decreased skeletal muscle *PDK2* expression. Although the precise function and induction of *PDK2* has not been fully elucidated, recent findings indicate that PDK isoenzyme is physiologically important to activate skeletal muscle PDC and pyruvate oxidation [40].

Expressions of enzymes coding for mitochondrial β-oxidation and its transcriptional coactivator PGC1α were not affected by HS or PF, whereas *ACAA1*, a β-ketothiolase involved in the final step of peroxisomal fatty acid degradation was upregulated in HS while tending to be downregulated in PF cows, pointing to increased long-chain fatty acid degradation in muscle peroxisomes.

The degradation of muscle protein may start in late gestation [41] or early lactation [39,42,43]. With the exception of *UBA52*, cows in early lactation exposed to heat did not change the expression of proteasomal genes despite increased *FOXO3* abundance. These data imply that HS in early lactation does not change skeletal muscle proteolysis on mRNA abundance despite reduced plane of nutrition. In line with this, HS has been shown to exert a greater effect on protein synthesis than protein breakdown in chickens [44,45].

Adaptation of hepatic gluconeogenesis in early lactation occurs in a different way than in late pregnancy (see above). In early lactation, *PC* mRNA abundance is greater in PF than HS cows. We conclude that when the supply of propionate for hepatic gluconeogenesis is limited, lactate and/or amino acid-derived pyruvate are used more readily as gluconeogenic substrates under thermoneutral PF conditions [46]. However, *PCK1* expression tended to be reduced in early lactation but to be increased in late pregnancy after heat exposure, indicating that the transcriptional regulation of this gene under hot temperatures depends on the physiological status. However, downregulation of *PCK1* in HSpp but not PFpp cows suggests less utilization of amino acid-derived pyruvate in HS that PF early lactating cows.

Furthermore, cows in early lactation, but not late-pregnant cows respond to HS by reducing the mRNA expression of *Atp5b*, a subunit of the ATP synthase. This result indicates that lactating cows reduce metabolic heat production by reducing ATP production, whilst oxidation through the respiratory chain complex I, as indicated by unchanged *ND2*, remained constant. Whether uncoupling of the electron transport chain leads to reduced ATP synthesis needs to be determined in future studies.

In early lactation, plasma proline and glycine concentrations were lower after HS compared to PF challenge, suggesting a more intensive utilization of these amino acids as precursors for gluconeogenesis during high ambient temperatures, but this assumption needs further investigation. Our results also contrast the findings by Tian et al. (2015), who described greater plasma glycine and proline concentrations of mid-lactating cows sampled during the summer compared to spring season, but this effect may also be attributed conditions others than heat stress [47].

Similar to the late gestation period, the mRNA abundances of urea cycle encoding enzymes are not affected by HS or PF in early lactation, but also in late gestation may be due to the slow responsiveness of these genes as described by Velez *et al*. [48]. Yet, plasma urea concentrations are almost doubled in HS lactating cows. Wheelock et al. (2010) suggested that the quick increase in plasma urea nitrogen would be due to increased hepatic deamination of amino acids [23]. Our present findings on unchanged urea cycle enzyme mRNA, however, argues against this assumption [23], but confirms that HS but not PF reduces kidney perfusion and enhances plasma water losses (also into milk), thus facilitating the accumulation of urea in plasma [16] without altered amino acid deamination.

Lactating cows are unable to mobilize adipose tissue and thus free fatty acids [23] and therefore cannot increase fat oxidation during heat stress [16]. Accordingly, mRNA abundance of enzymes involved in mitochondrial β-oxidation did not change after HS and PF. However, one week of HS showed lower *ACOX1* and *CAT* mRNA abundances of enzymes involved in peroxisomal β-oxidation, reducing peroxisomal heat production and oxidative stress. In cows that calved during hot summer compared to mild spring days, hepatic *ACOX1* was downregulated alike, but in addition to this, *CPT1A* and *PPARA* were also reduced, indicating a lower rate of total fatty acid oxidation in these HS cows [20].

In summary, metabolic adaptation to heat stress and reduced feed intake differ between late pregnancy and early lactation of dairy cows. In liver and skeletal muscle, a shift in substrate utilization is required to maintain gluconeogenesis for fetus development in late gestation and milk production in early lactation, through the reduction of endogenous heat production. This knowledge is crucial for urgently needed strategies mitigating heat stress, risks for diseases and economic losses, while sustaining animal well-being and performance.

## Supporting Information

S1 Table (PDF)
