# Supplementary material for: Metabolic Response to Heat Stress in Late-Pregnant and Early Lactation Dairy Cows: Implications to Liver-Muscle Crosstalk
Source: PLoS One. 2016 Aug 11;11(8):e0160912. doi: 10.1371/journal.pone.0160912 (PMC4981427; doi:10.1371/journal.pone.0160912)
Supplement: S1 Table — (PDF) [file pone.0160912.s001.pdf]

Supporting Information

S1 Table. Primer sequences and real-time PCR conditions.

Gene		Primer sequence (5' to 3')	GenBank accession no.	Product Size (base pairs)	Annealing (°C / s)	Efficiency
ACAA1	forward	TGTCCCTAGCTGACAGAGGG	NM_001034319.2	162	60	1.878 [l]
	reverse	GGGAAGCCAGTGCAAAGGTA			60	1.902 [m]
ACAA2	forward	TGAATGAAGCTTTTGCTCCTC	NM_001035342	224	60	1.876
	reverse	GATGATGACCGCAATTCCTT			60	
ACADVL	forward	AATGGAAGGTTTGGCATGGC	NM_174494	125	59	1.890 [l]
	reverse	TGGATCAGCCCAAAGTTGTG			59	1.899 [m]
ACOX1	forward	TTGTGGCGGACATGGCTATT	NM_001035289.3	148	60	1.877 [l]
	reverse	TGAGTGCACCTGGTCGTAAC			60	1.923 [m]
ACOX2	forward	AACATGCTGAATCGCTTCGC	NM_001102015.2	89	60	1.865
	reverse	ACCACCATGCTGAGGTAGTTAC			60	
ASL	forward	GTGGCCACTGGTGTCATCTCT	NM_001034428	58	62	1.910
	reverse	GAGCCCGTCCCATGTTCTC			62	
ATB5b	forward	TGCTTTATTGGGCAGAATCC	NM_175796	152	58	1.858
	reverse	GATCCGTCAAGTCATCAGCA			58	
CAPN1	forward	TTTCCAGCTGTGGCAGTTTG	NM_174259	89	59	1.908
	reverse	AGTGCACAAACACCAGCTTC			59	
CAPN2	forward	ACTGCCCAAACTGGAACACA	NM_001103086	148	60	1.894
	reverse	GTGAGTGTGTCAGGGGTCAG			60	
CAT	forward	TCACTCAGGTGCGGACTTTC	NM_001035386.2	162	60	1.872
	reverse	GGATGCGGGAGCCATATTCA			60	
CLN3	forward	TTCTGACTCCTTGGGACACA	NM_001075174	62	60	1.953
	reverse	CAACCTGCCCACCTATCAGT			60	
CPS	forward	GGTGGCTTGCTTTGGTGAAG	NM_001192258	188	60	1.888
	reverse	AGTCTGATGTGGCTTCCGTG			60	
CS	forward	TGGACATGATGTATGGTGG	NM_001044721	217	60	1.826
	reverse	AGCCAAGATACCTGTTCCTC			60	
EMD	forward	GCCCTCAGCTTCACTCTCAGA	NM_203361	100	59	1.915
	reverse	GAGGCGTTCCCGATCCTT			59	
FOXO3	forward	TCCAGACAAACGGCTCACTC	NM_001206083	172	60	1.877
	reverse	CTTGCCGGTTCCCTCATTCT			60	
GPT2	forward	TGTACCTGCAGACCCGGATA	NM_001193187	167	60	1.881
	reverse	TGGCATCGAGTTCGGAGATG			60	
HADH	forward	AACACCAATGACCAGCCAGA	NM_001046334.1	150	58	1.887 [l]
	reverse	TCACACAGCCTGACTGCTTC			58	1.924 [m]
HPCAL1	forward	CCATCGACTTCAGGGAGTTC	NM_001098964	99	60	1.907
	reverse	CGTCGAGGTCATACATGCTG			60	
LDHA	forward	TGGGGAGCATGGTGACTCTA	NM_174099.2	187	60	1.882 [l]
	reverse	AATGGCCCAGGATGTGTAGC			60	1.889 [m]
LDHB	forward	ACTTGGCATTCATCCCAGCA	NM_174100	90	60	1.912

	reverse	GCCACATTCACTCCACTCCA			60	
LRP10	forward	CCAGAGGATGAGGACGATGT	NM_001100371.1	139	60	1.896 [l]
	reverse	ATAGGGTTGCTGTCCCTGTG			60	1.908 [m]
ND2	forward	CATGCTCCGAAACTCTGACA	NM_174565.3	129	58	1.866
	reverse	GCATTTACACAGGCCCCTAA			58	
PC	forward	ACACCAACTACCCCGACAATG	NM_177946.4	353	60	1.867
	reverse	CAGCGGGAGGTCAGGGAAG			60	
PCK1	forward	CAACTCTCGCTTTTGTGCCC	NM_174737.2	122	60	1.869
	reverse	GGGGGACTCCTTTGGGTCTA			60	
PCK2	forward	CAAGGATGGGGAGCCTTGTG	NM_001205594.1	162	62	1.888
	reverse	CCTCCGAAGATGATGCCCTC			62	
PDK2	forward	AACCCCAGCAATGTTTCTGC	NM_001159481, NM_001075853.2	70	59	1.91
	reverse	TGGGTAGGTGTATGCACATGAG			59	
PDK4	forward	AGGTTTTCACGCTGTGGATG	NM_001101883	95	59	1.856
	reverse	AAAGTGCCATATGCGTACGC			59	
PPARA	forward	CAGAACAAGGAGGCGGAAGT	NM_001034036	215	60	1.893
	reverse	CCGTAGGCTACCAACATCCC			60	
PPARGC1A	forward	CCTGCATGAGTGTGTGCTCT	NM_177945.3	158	60	1.911
	reverse	GCAAAGAGGCTGGTCTTCAC			60	
UBB	forward	TCTGAGGGGTGGCTGTTAATTC	NM_174133	76	60	1.91
	reverse	ATGGCTAGAGTGCAGAAGGATG			60	
UBA52	forward	GGCAAGACCATCACCCTTGA	NM_001076363.2	220	62	1.876
	reverse	GGAGGGAAGGCTCGATGATG			62	

[l] liver

[m] skeletal muscle
